# Case Report: Sustained pubertal suppression for 68 months with a single histrelin acetate implant in central precocious puberty

**DOI:** 10.3389/fendo.2026.1951374

**Published:** 2026-10-09

**Authors:** Sofya Ilmer, Parissa Salemi

**Affiliations:** Department of Pediatrics, Division of Endocrinology and Metabolism, Northwell Health, New Hyde Park, NY, United States

**Keywords:** adult height prediction, central precocious puberty, GnRH agonist, histrelin acetate, implant duration, pubertal suppression

## Abstract

The subcutaneous histrelin acetate implant is FDA-approved for 12 months of continuous gonadotropin-releasing hormone (GnRH) agonist therapy in central precocious puberty (CPP). Published data demonstrated sustained suppression with a single implant for up to approximately 24 months; however, efficacy beyond this duration has not been reported. A 7 years 5 month old female patient presented with bilateral Tanner 2 breast development, a bone age of 10 years, and laboratory findings consistent with early central activation. Treatment was delayed 11 months due to disruptions in care during the COVID-19 pandemic, and a histrelin implant was placed at age 8 years 5 months. The patient was lost to follow-up and returned at nearly 14 years of age, approximately 68 months after the implant placement, with primary amenorrhea and pubertal arrest. Laboratory evaluation before implant removal showed suppressed but detectable gonadotropins with a prepubertal estradiol, consistent with sustained partial suppression of the hypothalamic-pituitary-gonadal (HPG) axis sufficient to arrest pubertal progression. Her predicted adult height (PAH) of 167.6 cm (66 inches) approximated her mid-parental height (MPH) of 166.4 cm (65.5 inches). This case demonstrates that a single histrelin acetate implant may maintain clinically meaningful pubertal suppression for at least 68 months. These findings support broader investigations into the pharmacokinetics of extended implant use, given implications for reducing surgical burden and healthcare costs in CPP management.

## 1 Introduction

Central precocious puberty (CPP) is defined as the premature activation of the hypothalamic-pituitary-gonadal (HPG) axis, resulting in the development of secondary sexual characteristics before age 8 years in girls and 9 years in boys. CPP is up to 15 times more common in girls than boys, with an estimated incidence of 29 per 100,000 girls in the United States (1). The condition is associated with diminished adult height potential due to accelerated skeletal maturation and early epiphyseal fusion, as well as adverse psychosocial outcomes (2, 3).

Gonadotropin-releasing hormone agonists (GnRHas) are the mainstay of treatment for CPP, providing sustained suppression of the HPG axis through continuous receptor desensitization. Among available formulations, the subcutaneous histrelin acetate implant (Supprelin LA) offers continuous drug delivery and is FDA-approved for 12 months of use. Although approved for annual replacement, published data have demonstrated sustained biochemical suppression with a single implant beyond 12 months. Ray et al. reported a retrospective series of 170 children in whom a single implant was left in place for an average of 24 months with maintained pubertal suppression (4). However, no published reports have documented efficacy beyond approximately 2 years from a single device.

We report a unique case of sustained pubertal suppression for approximately 68 months from a single histrelin implant in a patient with CPP—far exceeding any previously documented duration of efficacy from a single device. Because serum histrelin was not measured and gonadotropins remained detectable at presentation for removal, the effect is characterized as partial suppression of the HPG axis sufficient to arrest pubertal progression, rather than complete axis inhibition.

## 2 Case presentation

A girl initially presented in October 2019 at 7 years and 5 months of age with bilateral Tanner stage 2 breast development (**Table 1**). Her workup revealed a bone age of approximately 10 years. Laboratory evaluation revealed a luteinizing hormone (LH) of 0.159 mIU/mL (SI: 0.159 IU/L; Tanner stage 1 reference range, 0.02–0.18 mIU/mL [SI: 0.02–0.18 IU/L]), follicle-stimulating hormone (FSH) of 2.7 mIU/mL (SI: 2.7 IU/L; Tanner stage 1 reference range, 1.0–4.2 mIU/mL [SI: 1.0–4.2 IU/L]), and estradiol of 10 pg/mL (SI: 36.7 pmol/L; Tanner stage 1 reference range, 5.0–20 pg/mL [SI: 18.4–73.4 pmol/L]). At her follow up examination 3 months later in January 2020, she continued to show Tanner 2 examination of the breasts. Serial heights documented progressive growth acceleration: 132.4 cm on October 16, 2019, 135.5 cm on January 14, 2020, and 145.0 cm on July 15, 2020 (**Figure 1**), corresponding to an annualized height velocity of approximately 12.6 cm/year over the first interval and approximately 19 cm/year over the subsequent interval (approximately 16.8 cm/year across the full ~9-month pre-treatment period). This markedly exceeds the normal prepubertal velocity of approximately 5–6 cm/year and the velocity threshold of 7.0 cm/year reported to discriminate gonadotropin-dependent precocious puberty from premature thelarche in 6- to 8-year-old girls (5). In the context of sustained and progressive Tanner 2 breast development, markedly accelerated growth velocity, and advanced bone age (bone age–chronological age difference of approximately +2.6 years), the presentation was consistent with progressive central precocious puberty. Although the basal LH of 0.159 mIU/mL fell within the Tanner stage 1 reference range and FSH was the predominant gonadotropin — a pattern that overlaps with premature thelarche — a low or non-elevated basal LH does not exclude central precocious puberty in girls, in whom basal LH may confirm but not refute the diagnosis (6, 7). The diagnosis therefore rested on the composite clinical, auxological, and skeletal findings rather than on basal gonadotropins alone. GnRH stimulation testing could not be completed due to nationwide leuprolide acetate shortages at the time, therefore diagnosis of CPP was made based on clinical presentation and biochemical data. Two hand-wrist radiographs were obtained during the course of care: at initial presentation in October 2019 (**Figure 2**) and at the time of implant removal in 2026 (**Figure 3**). No radiograph was obtained at implant placement in September 2020 because of pandemic-related disruptions in care; the October 2019 bone age of approximately 10 years (bone age–chronological age difference approximately +2.6 years) was the skeletal-maturation basis for the treatment decision. Her PAH based on Greulich and Pyle was 160.0–167.6 cm (63–66 inches) relative to her MPH of 166.4 cm (65.5 inches).Due to COVID Pandemic, last visit in July 2020 prior to patient being lost to follow up was by televideo visit. Following an unremarkable brain MRI in January 2020, a histrelin implant was placed in September 2020. Although the family was counseled on the need for annual re-evaluation and implant replacement, they later reported a misunderstanding that the implant would “dissolve on its own,” which contributed to the prolonged interval before returning for care when primary amenorrhea prompted re-evaluation.

**Table 1:** Clinical timeline of care.

Time point	Age	Tanner stage	LH (mIU/mL)	FSH (mIU/mL)	Estradiol (pg/mL)	Bone age (yr)	Height, cm (in)	PAH, cm (in)
Diagnosis (October 2019)	7y 5mo	B2	0.159	2.7	10	~10	132.4 (52.1)	160.0-167.6 (63–66)
Implant placement (September 2020)	8y 5mo	—	—	—	—	—	—	—
Return visit (April 2026)	13y 11mo	B1 (regressed glandular tissue)	0.254	2.8	2.3	13.5–14	164.1 (64.6)	167.6 (66)
4 weeks after Implant removal (June 2026)	14y 1mo	–	0.707	8.3	31	–	–	–

LH, luteinizing hormone; FSH, follicle stimulating hormone; PAH, predicted adult height.

**Figure 1:**
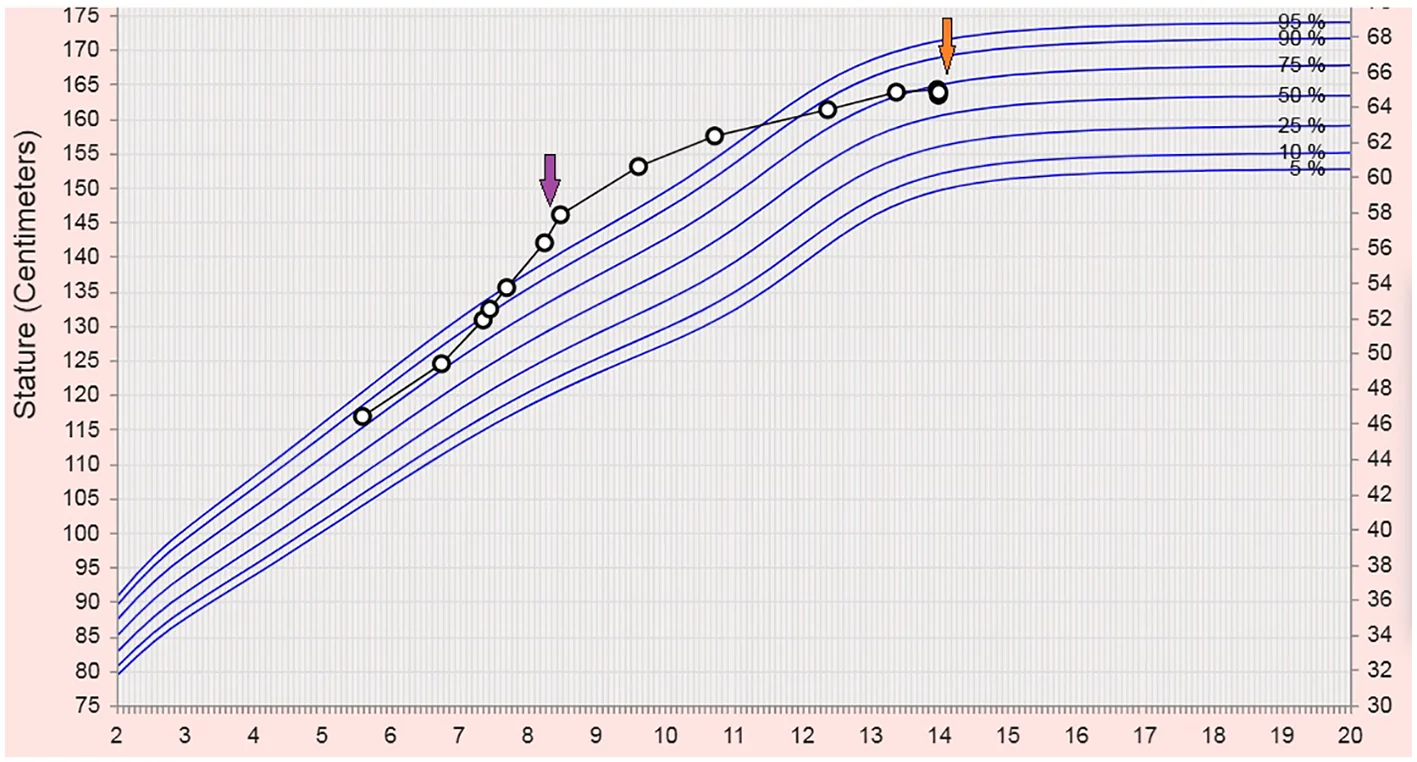
Patient’s height growth chart. Purple arrow marks histrelin acetate implant placement. Orange arrow marks histrelin acetate implant removal.

**Figure 2:**
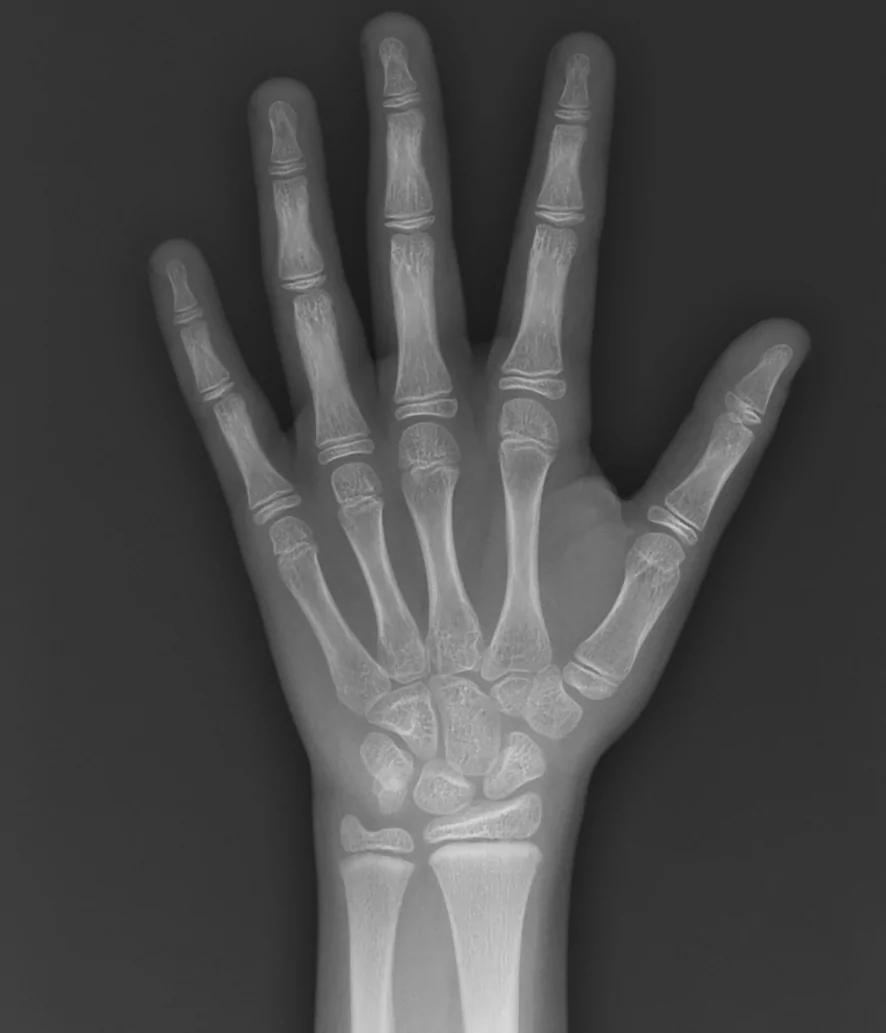
Bone age at diagnosis (October 2019): left hand–wrist radiograph at chronological age 7 years 5 months showing a bone age of approximately 10 years (Greulich and Pyle).

**Figure 3:**
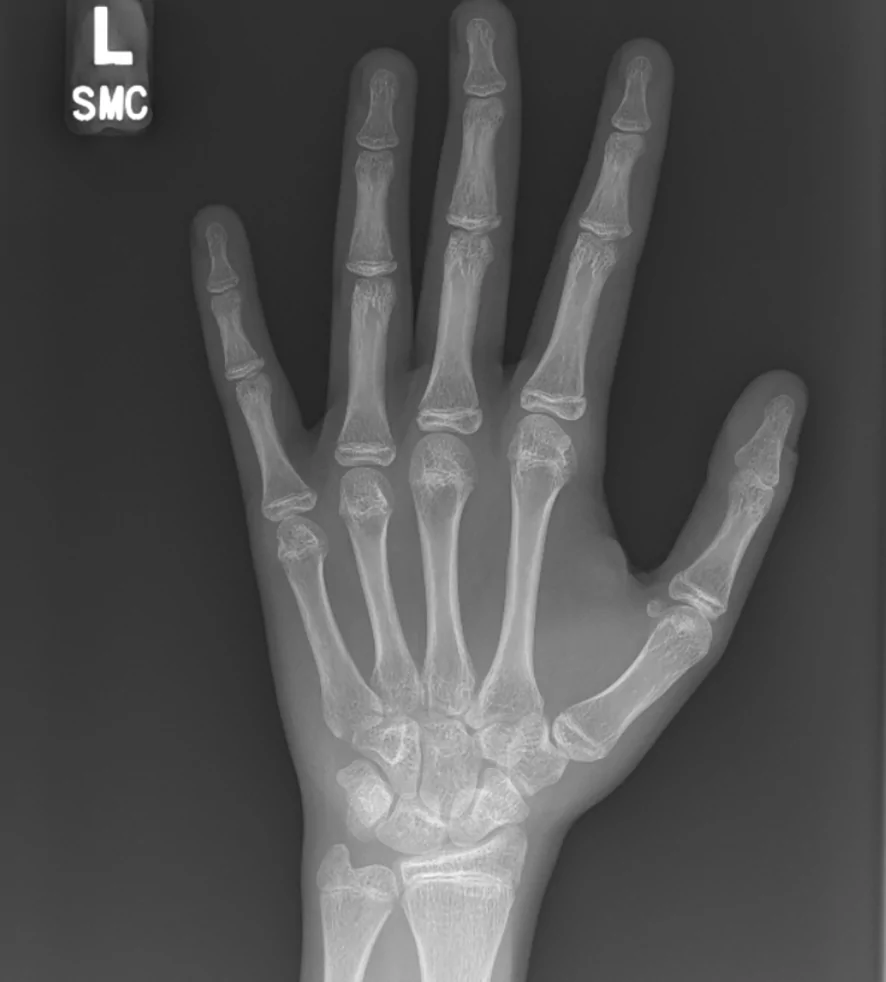
Bone age at return visit (2026): left hand–wrist radiograph at chronological age 13 years 11 months showing a bone age of 13.5–14 years (Greulich and Pyle).

## 3 Timeline

**Table 1** presents the clinical timeline of care, including relevant data from each episode of care.

### 3.1 Therapeutic intervention

A histrelin acetate implant (Supprelin LA) was placed subcutaneously in September 2020 at age 8 years 5 months. The implant contains 50 mg of histrelin acetate within a nonbiodegradable, diffusion-controlled hydrogel polymer reservoir, designed to deliver approximately 65 mcg/day (8). The implant remained *in situ* for approximately 68 months without replacement.

### 3.2 Reassessment at return visit

The patient was subsequently lost to endocrine follow-up and returned to pediatric endocrinology at nearly 14 years of age with primary amenorrhea. On examination, she exhibited a Tanner 1 breast evaluation. Laboratory evaluation revealed an LH of 0.254 mIU/mL (SI: 0.254 IU/L; Tanner stage 1 reference range, 0.02–0.18 mIU/mL [SI: 0.02–0.18 IU/L]), FSH of 2.8 mIU/mL (SI: 2.8 IU/L; Tanner stage 1 reference range, 1.0 – 4.2 mIU/mL [SI: 1.0–4.2 IU/L]), and estradiol of 2.3 pg/mL (SI: 8.4 pmol/L; Tanner stage 1 reference range, 7.0–60 pg/mL [SI: 18.4–73.4pmol/L]). Although LH remained detectable above Tanner 1 reference range, the prepubertal estradiol together with the absence of menarche and lack of pubertal progression indicated sustained partial suppression of the HPG axis. Her height was 164.1 cm (64.6 inches), and bone age was 13.5–14 years, corresponding to a BA−CA difference of approximately 0 to +0.1 years—a marked narrowing from the +2.6 years at diagnosis—with a PAH of 167.6 cm (66 inches) by Greulich and Pyle (**Table 1**).

### 3.3 Follow-up and outcomes

The implant was removed on May 11, 2026. The operative report described the implant as “quite scarred into the adjacent soft tissues in the subcutaneous tissue.” During removal, the implant fractured into two pieces but was removed in its entirety (**Figure 4**). The explanted implant measured approximately 4 cm (1.5 inches) in total length (**Figure 5**). The patient’s PAH of 167.6 cm (66 inches) approximated her MPH of 166.4 cm (65.5 inches) (**Table 1**). Laboratory evaluation 4 weeks after implant removal showed LH 0.707 mIU/mL (SI: 0.707 IU/L), FSH 8.3 mIU/mL (SI: 8.3 IU/L), and estradiol 31 pg/mL (SI: 113.8 pmol/L), consistent with early recovery of the HPG axis. Final adult height, timing of menarche, and skeletal outcomes remain to be determined.

**Figure 4:**
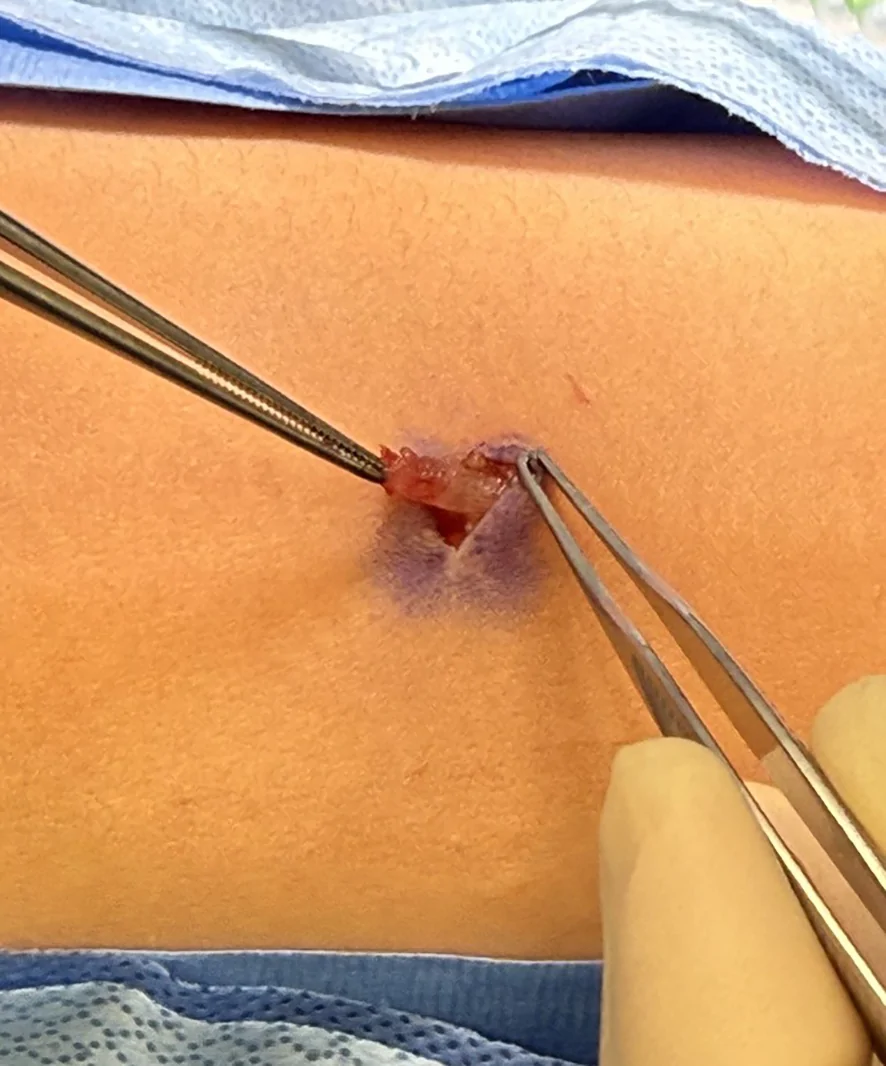
Intraoperative photograph demonstrating the histrelin acetate implant site during surgical removal at 68 months post-insertion. The implant was noted to be densely adherent to surrounding subcutaneous tissues with significant fibrotic encapsulation.

**Figure 5:**
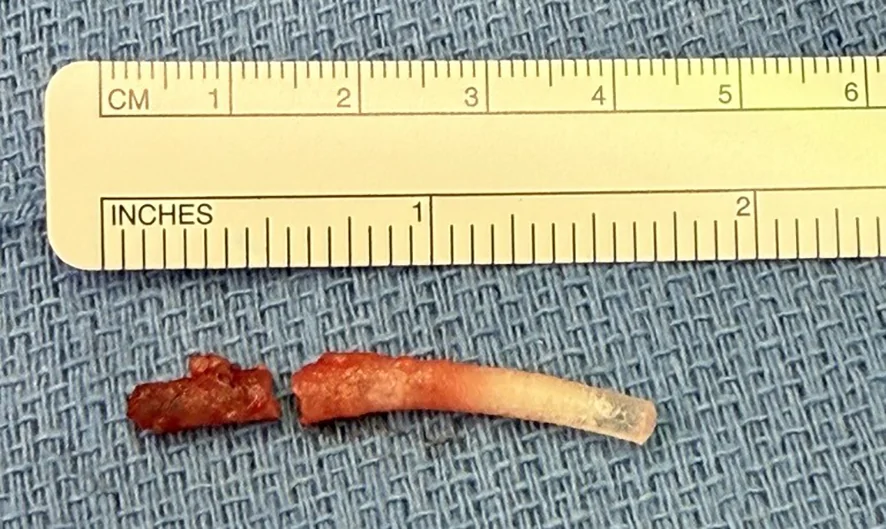
Explanted histrelin acetate implant after 68 months in situ, shown in two fragments following removal.

## 4 Discussion

This case demonstrates sustained partial suppression of the HPG axis for approximately 68 months from a single histrelin acetate implant, far exceeding the FDA-approved 12-month duration (4). The Supprelin LA implant contains 50 mg of histrelin acetate within a nonbiodegradable, diffusion-controlled hydrogel polymer reservoir, designed to deliver approximately 65 mcg/day (8). At this release rate, only approximately 24 mg (~47%) of the total drug content would be expected to be released over 12 months, leaving a substantial reservoir within the implant. A fraction of the original release rate may suffice to maintain pituitary desensitization, providing a plausible pharmacokinetic mechanism for the extended effect (8). Because serum histrelin was not measured and gonadotropins remained detectable at 68 months, the effect is best interpreted as partial suppression adequate to arrest pubertal progression rather than complete axis inhibition. The dense fibrotic encapsulation observed at the time of removal (**Figures 2**, **3**) may have further contributed to prolonged efficacy by maintaining close apposition of the implant to vascularized tissue.

These observations extend prior reports. Ray et al. described maintained pubertal suppression in 170 children with a single implant left in place for an average of 24 months, and Silverman et al. demonstrated sustained suppression with sequential annual implants over up to 6 years (4, 9). In the prostate cancer literature, a single histrelin implant maintained testosterone suppression for up to 30 months, and sequential annual implants sustained suppression for over 4 years, further supporting the durability of the hydrogel delivery system (10, 11). The present case is the first to document clinically meaningful suppression from a single implant for nearly 6 years.

The height trajectory is reassuring but not yet definitive. The BA−CA difference narrowed from approximately +2.6 years at diagnosis to near zero at removal, indicating that continued suppression slowed skeletal maturation relative to chronological age and likely preserved height potential. The patient’s PAH approximated her MPH, consistent with the known height benefit of GnRHa therapy in CPP (2, 3, 6, 9, 12, 13). However, whether treatment extending beyond the optimal bone age for discontinuation confers additional benefit or compromises the pubertal growth spurt cannot be determined until final height is attained, since post-treatment linear growth is not predictable. At return, the bone age of 13.5–14 years suggests the height benefit had likely plateaued and the timing of removal was appropriate.

Reproductive recovery after GnRHa therapy is generally favorable: the phase 3 histrelin extension study showed full HPG axis recovery within 6 months of removal, and longitudinal cohorts report normal menses and successful pregnancies (2, 9, 14–16). GnRHa therapy is associated with reversible reductions in bone mineral density during treatment, with preserved subsequent accrual; nonetheless, the unusually prolonged estrogen deprivation in this case warrants bone density assessment following resumption of gonadal function (6, 14, 15, 17).

The principal practical implication is that a single implant may sustain meaningful suppression well beyond the annual replacement schedule, with potential to reduce surgical procedures, cost, and treatment burden—particularly where subspecialty access is limited.

This report has limitations inherent to a single case. First, the diagnosis of CPP was established on clinical and basal biochemical grounds because GnRH stimulation testing was unavailable during a national leuprolide shortage; a stimulation-confirmed diagnosis would have strengthened diagnostic certainty. Second, serum histrelin and residual implant drug content were not measured, so the quantity of drug utilized at 68 months is unknown. Third, no interval monitoring occurred during the 68 months the implant was in place, so the trajectory of suppression over time cannot be characterized, and the 68-month values may represent a nadir of drug release that was still sufficient for partial suppression. Finally, auxological data and bone densitometry at menarche and final height are not yet available. Longitudinal follow-up of this patient, including auxological data, bone densitometry, and reproductive outcomes at menarche and at attainment of final height, is planned and will be reported when available. In addition, bone age was documented at only two time points (presentation and removal), with no radiograph at implant placement, and basal gonadotropins at presentation showed a non-elevated LH with FSH predominance; the diagnosis of progressive CPP therefore rested on accelerated growth velocity and advanced bone age rather than on unequivocal basal biochemical activation.

## 5 Conclusions

A single histrelin acetate implant maintained partial but clinically meaningful suppression of the HPG axis for approximately 68 months, which is far beyond the FDA-approved 12-month duration and the previously reported maximum of approximately 24 months from a single device, arresting pubertal progression until removal, with a PAH that approximated the patient’s MPH. The large residual drug reservoir provides a pharmacokinetic basis for efficacy beyond the labeled duration. Two clinical implications can be considered: first, extended single-implant use merits prospective study of its pharmacokinetics and safety, as it could reduce surgical burden and cost in CPP management; and second, families should be counseled in writing, with documentation in the medical record, about the mandatory schedule for follow-up visits and timely implant removal, to prevent inadvertently prolonged retention such as occurred here.

## 6 Patient perspective

The patient’s mother reported that she had believed the implant would resorb on its own and therefore did not return for scheduled follow-up. At the return visit, she expressed concern about the prolonged duration of suppression and about her daughter’s lack of pubertal progression. After implant removal, follow-up laboratory testing showed rising gonadotropin and estradiol levels; the mother was informed that these results were consistent with early recovery of the HPG axis.

## Data Availability

The original contributions presented in the study are included in the article/supplementary material. Further inquiries can be directed to the corresponding author.

## References

[1] Fuqua JS . Treatment and outcomes of precocious puberty: an update. J Clin Endocrinol Metab. (2013) 98:2198–207. doi: 10.1210/jc.2013-1024 23515450

[2] Zevin EL Eugster EA . Central precocious puberty: a review of diagnosis, treatment, and outcomes. Lancet Child Adolesc Health. (2023) 7:886–96. doi: 10.1016/s2352-4642(23)00237-7 37973253

[3] Carel J-C Léger J . Precocious puberty. N Engl J Med. (2008) 358:2366–77. doi: 10.1056/nejmcp0800459 18509122

[4] Ray LA Eckert GJ Eugster EA . Long-term experience with the use of a single histrelin implant beyond one year in patients with central precocious puberty. J Pediatr Endocrinol Metab. (2023) 36:309–12. doi: 10.1515/jpem-2022-0557 36625262

[5] Varimo T Huttunen H Miettinen PJ Kariola L Hietamäki J Tarkkanen A . Precocious puberty or premature thelarche: analysis of a large patient series in a single tertiary center with special emphasis on 6- to 8-year-old girls. Front Endocrinol (Lausanne). (2017) 8:213. doi: 10.3389/fendo.2017.00213 28878739 PMC5572337

[6] Latronico AC Roberts SA Alonzo M Argente J Canton APM Carel J-C . Central precocious puberty: an Endocrine Society clinical practice guideline. J Clin Endocrinol Metab. (2026) 111:2101–44. doi: 10.1210/clinem/dgag168 42287186

[7] Howard SR . Interpretation of reproductive hormones before, during and after the pubertal transition—identifying health and disordered puberty. Clin Endocrinol. (2021) 95:702–15. doi: 10.1111/cen.14578 34368982 PMC9291332

[8] SUPPRELIN LA- histrelin acetate implant Drug Label. Malvern, PA: Endo Pharmaceuticals. (2025). Available online at: https://www.accessdata.fda.gov/drugsatfda_docs/label/2025/022058s020lbl.pdf.

[9] Silverman LA Neely EK Kletter GB Lewis K Chitra S Terleckyj O . Long-term continuous suppression with once-yearly histrelin subcutaneous implants for the treatment of central precocious puberty: a final report of a phase 3 multicenter trial. J Clin Endocrinol Metab. (2015) 100:2354–63. doi: 10.1210/jc.2014-3031 25803268 PMC4504932

[10] Shore N Cookson MS Gittelman MC . Long-term efficacy and tolerability of once-yearly histrelin acetate subcutaneous implant in patients with advanced prostate cancer. BJU Int. (2012) 109:226–32. doi: 10.1111/j.1464-410X.2011.10370.x 21851539

[11] Chertin B Spitz IM Lindenberg T Algur N Zer T Kuzma P . An implant releasing the gonadotropin hormone-releasing hormone agonist histrelin maintains medical castration for up to 30 months in metastatic prostate cancer. J Urol. (2000) 163:838–44. doi: 10.1016/s0022-5347(05)67816-0 10687989

[12] Luo X Liang Y Hou L Wu W Ying Y Ye F . Long-term efficacy and safety of gonadotropin-releasing hormone analog treatment in children with idiopathic central precocious puberty: a systematic review and meta-analysis. Clin Endocrinol (Oxf). (2021) 94:786–96. doi: 10.1111/cen.14410 33387371 PMC8248422

[13] Vargas Trujillo M Dragnic S Aldridge P Klein KO . Importance of individualizing treatment decisions in girls with central precocious puberty when initiating treatment after age 7 years or continuing beyond a chronological age of 10 years or a bone age of 12 years. J Pediatr Endocrinol Metab. (2021) 34:733–9. doi: 10.1515/jpem-2021-0114 33856747

[14] Guaraldi F Beccuti G Gori D Ghizzoni L . Management of endocrine disease: long-term outcomes of the treatment of central precocious puberty. Eur J Endocrinol. (2016) 174:R79–87. doi: 10.1530/eje-15-0590 26466612

[15] Pasquino AM Pucarelli I Accardo F Demiraj V Segni M Di Nardo R . Long-term observation of 87 girls with idiopathic central precocious puberty treated with gonadotropin-releasing hormone analogs: impact on adult height, body mass index, bone mineral content, and reproductive function. J Clin Endocrinol Metab. (2008) 93:190–5. doi: 10.1210/jc.2007-1216 17940112

[16] Martinerie L de Mouzon J Blumberg J di Nicola L Maisonobe P Carel JC . Fertility of women treated during childhood with triptorelin (depot formulation) for central precocious puberty: the PREFER study. Horm Res Paediatr. (2020) 93:529–38. doi: 10.1159/000513702 33774631 PMC8686727

[17] Guo N Zhou F Jiang X Yang L Ma H . The effect of gonadotropin-releasing hormone analog treatment on the endocrine system in central precocious puberty patients: a meta-analysis. J Pediatr Endocrinol Metab. (2024) 37:197–208. doi: 10.1515/jpem-2023-0480 38235550

